# Breastfeeding and Complementary Feeding Practices of Children with Stunting in Guatemala

**DOI:** 10.5334/aogh.5266

**Published:** 2026-08-06

**Authors:** Stephen Alajajian, Charis Eirené Gudiel de León, Lilian Carolina Ajú Batz, Peter Rohloff

**Affiliations:** 1 Wuqu’ Kawoq | Maya Health Alliance, Tecpán, Chimaltenango, Guatemala; 2 Brigham and Women’s Hospital, Boston, MA, USA

**Keywords:** stunting, chronic malnutrition, linear growth, breastfeeding, complementary feeding, Guatemala, clinical nutrition, low‑ and middle‑income countries

## Abstract

*Background:* Stunting is a pervasive issue in low‑ and middle‑income countries, reflecting biological processes that adversely affect childhood cognitive development and increase the risk of chronic disease in adulthood. Nutritional intake is an important causative factor in stunting. Understanding the nutritional intake patterns of children with stunting can help inform nutrition program development.

*Objective:* To characterize breastfeeding and dietary patterns from a clinical cohort of children with stunting in Guatemala and identify factors associated with linear growth.

*Methods:* We included children with at least one diet record and one length/height‑for‑age z‑score below −2 from ages 0–5 years. We excluded children enrolled in complex care for severe non‑nutritional illness and records from prior to 6 months of age. We described adherence to World Health Organization infant and young child feeding indicators upon program enrollment. We longitudinally characterized breastfeeding and complementary feeding patterns of the cohort using generalized additive mixed modeling. We identified and quantified associations between various nutritional factors and linear growth using linear mixed effects models.

*Results:* The final analytical dataset included 19,476 patient encounters from 2,352 children. Most children did not meet World Health Organization standards for dietary adequacy upon enrollment in the program. Dietary intakes were predominantly carbohydrate‑based. The factors most strongly associated with linear growth were food insecurity (negatively associated), portion size, and continued breastfeeding from 12 to 23 months. Adherence to the infant and young child feeding indicators was positively associated with linear growth. The intake of most food groups was also positively associated with linear growth.

*Conclusions:* These findings suggest that the nutritional focus of interventions should be on adequate dietary diversity, earlier introduction and higher frequency of the less frequently consumed food groups, age‑appropriate portion sizes, and breastfeeding through 2 years of age. Public policy measures to address food insecurity are also necessary.

## Introduction

Childhood stunting, a condition in which children fail to reach their genetic height potential, is a pervasive issue in countries where poverty is widespread [1]. The biological processes underlying stunting adversely affect childhood cognitive development and increase the risk of chronic disease development later in life [2]. Although height itself is not the causal determinant of poor development, serving rather as a marker for nutritional status, environmental exposures, and underlying biological processes, addressing the root causes of stunting is crucial for allowing children to reach their full developmental potential [3]. The critical window of intervention for stunting is typically defined as the roughly 1000‑day period between conception and 2 years of age, but children may experience catch‑up growth beyond this period, and even in the absence of catch‑up growth, developmental outcomes may still improve if the underlying causal factors resolve [3, 4].

Stunting has multiple levels of causation, from broader factors like poverty, healthcare infrastructure, and food systems to more immediate factors like fetal growth restriction, childhood illness, hygiene, nutritional intake, digestive health, and systemic inflammation [1, 5]. Prolonged poor quality of nutritional intake, even if energy needs are met, is an important contributor to stunting. Stunting is often conceptualized as representing micronutrient deficiencies, as opposed to wasting, a form of acute malnutrition that is typically understood to be caused by insufficient energy intake. However, the two conditions are related and share common causes [2, 5]. The World Health Organization (WHO) has released dietary guidelines for infants and young children that define what constitutes an adequate dietary intake [6]. It is important to note that optimal nutrient absorption requires a healthy digestive system and gut microbial ecology.

Guatemala has a stunting prevalence of 46.5% among children under the age of 5, which is significantly higher than its neighbors and one of the highest in the world [1, 7]. The Indigenous Maya population is affected the most with the highest rates of stunting being seen in rural and predominantly Indigenous Maya areas like the departments of Quiché, Totonicapán, and Huehuetenango [7]. These same departments are also considered food insecurity crisis areas [8], suggesting an important link between childhood stunting and food insecurity. To better address childhood stunting in Guatemala, a multifaceted approach is needed. Given the importance of nutrition, understanding the dietary patterns of children with stunting, and how closely they conform to or deviate from WHO recommendations, is helpful for developing nutrition programs that focus on the right things.

Research suggests that dietary patterns of Indigenous Maya children with stunting in Guatemala tend to be characterized by frequent breastfeeding, diets rich in grain‑based beverages called *atoles* and other carbohydrates, limited dietary diversity with most protein coming from plant‑based sources, and deficient intakes of key nutrients including iron, zinc, choline, potassium, and omega‑3 fats [9–11]. A cross‑sectional study of preschool‑aged children in Mexico found that dietary patterns characterized predominantly by 1) fruit and vegetable intake and 2) corn‑based and other traditional foods were associated with higher prevalence of stunting, whereas a Western dietary pattern characterized predominantly by animal flesh foods, high‑fat foods, and high‑sugar foods and beverages was associated with lower stunting prevalence but higher overweight prevalence [12].

To our knowledge, no published studies from Guatemala or Latin America have characterized nutritional intake patterns of children with stunting using longitudinal data. All have used cross‑sectional data. One of the benefits of longitudinal analysis, in addition to understanding chronological trends, is the ability to quantify associations between different dietary factors and linear growth while considering the individual growth trajectory of each child. Such a design provides stronger evidence for causal effects than a cross‑sectional design [13]. In light of this, we conducted a retrospective chart review of longitudinal diet recall and growth data from a clinical cohort of children enrolled in a nutrition program for stunting in rural Guatemala to characterize breastfeeding and dietary patterns and to identify factors associated positively or negatively with linear growth.

## Methods

### Overview

We conducted a longitudinal observational study in a clinical cohort of children enrolled in a nutrition program to address stunting in rural Guatemala, primarily in the departments of Chimaltenango, Sololá, Suchitepéquez, and Quetzaltenango. The nutrition program enrolls children with stunting or at risk for stunting between the ages of 6 months and 5 years and consists of a minimum of six monthly home visits in which 24‑hour diet recalls and anthropometric measurements are taken. Core interventions of the nutrition program include monthly weight and height monitoring, family food rations, nutrition counseling, micronutrient supplementation, and childhood illness management. The interventions are provided through individualized home visits that take place in the native language of the participants. For the study, data were extracted from electronic medical records (EMR). We made use of data on child anthropometry and data from 24‑hour dietary recalls that were collected monthly during home visits for children as a part of the clinical workflow of the nutrition program. Ethics approval for the study was obtained from the Wuqu’ Kawoq (WK) Institutional Review Board (IRB) (WK‑2025‑001). Informed consent was waived as the study was expected to carry minimal risk and to be infeasible without the waiver.

### Inclusion and exclusion criteria

We included children in the medical record who had at least one diet record from under 5 years of age and at least one length/height‑for‑age z‑score (HAZ) below −2 standard deviations (classifying the child as having stunting based on the WHO definition) using a height measurement from prior to 5 years of age. We excluded children who were enrolled in Maya Health Alliance’s complex care program for individuals with severe non‑nutritional illness (e.g., inborn errors of metabolism). We additionally excluded individual records for which 50% or more of the dietary intake data were missing and for which the child was under 6 months of age. We chose to exclude records prior to 6 months of age because these records were rare and typically represented cases in which mothers had breastfeeding difficulties; thus, they were not representative of the typical child in the cohort and would have biased the sample if included. Given that this was a retrospective chart review, study size was limited to the number of children in the medical record who met inclusion and exclusion criteria.

### Data extraction and initial cleaning

Data extraction occurred on August 12, 2025. Data for relevant variables were extracted from the clinical records of children who had at least one diet recall dated to under five years of age and who were not in Maya Health Alliance’s complex care program. All clinical records were considered since Maya Health Alliance’s initial implementation of OpenMRS in March 2011. Only variables collected when the child was under five years of age were extracted. Data were extracted one variable at a time in batches using read‑only standard query language (SQL) and were stored on a secure cloud server and a local encrypted hard drive for analysis. Data cleaning was performed in R version 4.4.3. Each child was assigned a unique study ID. Records for which 50% or more of the dietary intake data were missing were removed. To prevent reidentification, date of birth was randomly shifted by 1–5 days in either direction prior to deriving age in months at each encounter. All remaining patient identifiers were removed from the dataset. Observations corresponding to children without any HAZ below −2 standard deviations were removed, consistent with inclusion criteria. Implausible values for key variables were made NA (not available). These included dietary intake variables for which the food group in question was recorded as having been consumed 10 or more times the previous day and HAZ values below −6 or above 6. Length/height‑for‑age z‑score values were also made NA if greater than zero and inconsistent with the trend of longitudinal z‑scores for that child in a way that was obviously erroneous, which occurred in 10 instances. Patient encounters for which child age was less than 6 months were also removed.

### Variables and measurement

Available variables from the clinical workflow of the nutrition program included: age at encounter, language, ethnicity, region, sex, birthweight, height, weight, height‑for‑age z‑score, height‑for‑age difference (HAD), moderate acute malnutrition (MAM) and severe acute malnutrition (SAM) presence, food insecurity level, number of siblings, and dietary variables, including WHO Infant and Young Child Feeding (IYCF) indicators.

Height‑for‑age z‑score, representing the number of standard deviations from the population reference median, was derived from height, sex, and age at encounter using the *anthro* package. The default algorithm was used to assign recumbent versus standing height. Height‑for‑age difference, representing the difference in centimeters between measured height and the population reference median, was derived from height, sex, and age at encounter using the LMS2z function from the *sitar* package. It was only calculated when HAZ was available after the removal of implausible values. Negative values for HAD corresponded to heights below the WHO growth standard median height. Height/length in centimeters and weight in kilograms were measured monthly via stadiometer and hanging scale, respectively, during the nutrition program home visits.

MAM and SAM presence was derived from weight‑for‑height z‑scores (WHZ), where a z‑score of −2 was the cutoff for MAM and a z‑score of −3 was the cutoff for SAM. Birthweight had many implausibly high values, suggesting that in those instances it may have been recorded in pounds (lbs) rather than kilograms (kg). Therefore, recorded birthweights above 4.5 were assumed to be in lbs and were converted to kg by dividing by 2.2. Birthweights above 10 were assumed to be erroneous and were made NA.

Sociodemographic and birth‑history‑related variables were collected verbally through an intake form upon enrollment in the program. Language and ethnicity, open text fields in the EMR, were dichotomized into (1) Spanish and Mayan language and (2) Indigenous and Ladino/Mestizo, respectively. Food insecurity level was determined by whether the parent or caregiver could provide their child daily with (1) an egg; (2) fruit; and (3) vegetables. Those who could provide all three daily were classified as “low,” those who could not give any of the three were classified as “high” and those who could give one or two were classified as “moderate.”

Dietary variables included the following 24‑hour recall intake frequency items from a clinical tool designed to evaluate dietary quality using WHO indicators: broth, carbohydrates, coffee/tea, condiments, natural or store‑bought fruit drinks, soda, eggs, animal flesh foods, fats and oils, refined foods with added sugar, fruits, and vegetables with vitamin A, other fruits and vegetables, *atoles* legumes, juice, dairy, milk/formula, soup, and sugar water. A “sweet beverage” variable was created that represented the sum of the intake frequencies of fruit drink, juice, sugar water, and soda. Additional 24‑hour recall‑related dietary variables included meal frequency, whether breastfeeding, breastfeeding frequency, whether solid, semi‑solid, or soft food was consumed, portion size at meals, number of food groups consumed (including breast milk), micronutrient supplement frequency (per week), continued breastfeeding between 12 and 23 months, and continued breastfeeding at 24 months or beyond. Aggregated variables were created for animal‑source foods (meat, fish, eggs, and dairy), all fruits and vegetables (vitamin A‑rich or other), and carbohydrates and sugars (*atoles*, other carbohydrates, refined foods with added sugar, and sweet beverages). The purpose of these aggregations was to make comparisons to WHO complementary feeding guidelines.

Several WHO IYCF indicators were also derived: minimum dietary diversity (MDD), minimum meal frequency (MMF), minimum acceptable diet (MAD), zero fruit or vegetable consumption, egg and/or flesh food consumption, sweet beverage consumption, and unhealthy foods consumption. These variables were created only for children between the ages of 6 and 23 months per WHO IYCF guidelines. MDD was met when five of eight groups (including breastmilk) had been consumed. Whether MMF was met depended on age at encounter, breastfeeding status, meal frequency and milk/formula intake frequency, and WHO guidelines were followed for the calculation. Minimum acceptable diet was met only if MDD and MMF were both met. Sweet beverage consumption represented the sum of the intake frequencies of fruit drinks, juice, sugar water, and soda. Unhealthy food consumption represented only the intake frequency of refined foods with added sugar, as no other unhealthy food class was recorded as a separate category.

### Statistical methods

Descriptive statistics of baseline characteristics were presented for the clinical cohort as a whole and for each department using the *gtsummary* package. Counts and percentages were presented for categorical variables, and medians alongside first and third quartiles were presented for numerical variables.

Generalized additive mixed modeling (GAMM) was used to predict frequency of breastfeeding and frequency of intake of different food groups from 6 to 60 months with 95% confidence intervals. The *gamm* from the *mgcv* package was used for modeling. The random effects structure included a random intercept for the child to account for inter‑individual correlation, and a negative binomial distribution was specified for the model given that the response variables were count variables that often exhibited overdispersion [14]. Predicted intake frequencies with confidence intervals were graphed using *ggplot*. The predicted data were used to identify, for each food group, the age at which predicted frequency of intake first reached thresholds of 0.5 (every other day consumption) and 1 (daily consumption). These ages were then graphed onto timelines to show the relative order in which the intake of different food groups first reached each threshold. A separate GAMM analysis was performed for the aggregated food groups to compare patterns against WHO complementary feeding guidelines.

Linear mixed effects (LME) models were used to quantify relationships between breastfeeding and dietary factors and linear growth. Missing data were first imputed using multivariate imputation by chained equations (MICE) with the mice package. In the base model, HAD (in centimeters) was the outcome of interest, and the confounders age, sex, and birthweight were included as fixed effects. For random effects, child ID and department were included as random intercepts with child ID nested inside department. A separate model was created for each breastfeeding or dietary factor of interest wherein that factor was added as a fixed effect to the base model. Dietary predictors included food insecurity, portion size, meal frequency, number of food groups consumed and intake frequency of carbohydrates, eggs, meat and fish, fats and oils, refined foods with added sugar, *atoles* legumes, fruits and vegetables with vitamin A, other fruits and vegetables, dairy products, sweet beverages, and micronutrient supplements.

The same process was repeated for variables that required specific age ranges: WHO IYCF indicators (6–23 months) and continued breastfeeding (12–23 months) and continued breastfeeding (24+ months). For each of these, the dataset was appropriately age‑restricted before performing multivariate imputation. For the WHO IYCF models, department was included as a fixed effect rather than a random effect due to convergence issues with nested random effects. Coefficients for dietary and breastfeeding fixed effects predictors were taken to represent the strength of the relationship, positive or negative, between that factor and linear growth. These were visualized with a forest plot using *forestploter* and *ggplot*. In a separate analysis, an interaction term between the factor of interest and age was added to each model to allow the model to account for varying relationships between each factor and linear growth across ages. These were visualized with margins plots using the *margins* package along with *ggplot*.

## Results

**Figure 1:**
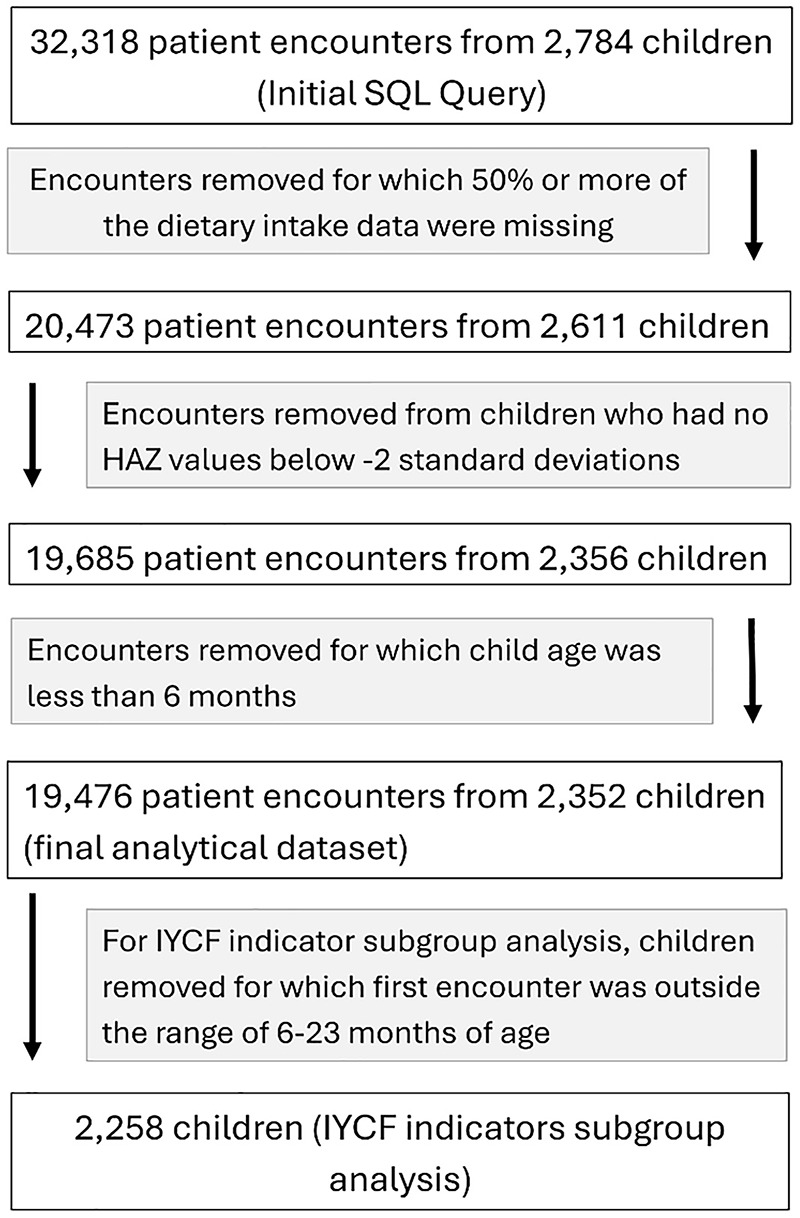
Flowchart of arrival at final analytical dataset.

The final analytical dataset included 19,476 patient encounters from 2,352 children, as shown in the flowchart in Figure 1. The median number of encounters per child was 9 (Q1–Q3: 8–9 encounters). The median encounter age was 17.9 months (Q1–Q3: 13.6–22.6 months). Missingness was highest for ethnicity, language, micronutrient intake frequency, and breastfeeding frequency and was approximately 10% for weight and height‑related variables (see Supplementary Table 1).

Descriptive statistics for the clinical cohort are shown in Table 1. Most caregivers identified as Indigenous and spoke Mayan languages. About three‑quarters of households were classified as having moderate or high food insecurity. The median age at enrollment was approximately 14 months of age and the median HAZ at enrollment corresponded to moderate stunting. All but one child were from four departments: Chimaltenango, Sololá, Suchitepéquez, and Quetzaltenango. Severity of stunting was similar across departments. Relative to other departments, Suchitepéquez had higher food insecurity and higher acute malnutrition prevalence, while Quetzaltenango had lower acute malnutrition prevalence. Dietary and breastfeeding patterns across geography, as well as age at enrollment and malnutrition status, are depicted visually in Supplementary Maps.

**Table 1:** Description upon program enrollment of a clinical cohort of children with stunting in Guatemala, overall and by department.

CHARACTERISTIC	CHIMALTENANGO *N* = 942^1^	SOLOLá *N* = 406^1^	SUCHITEPéQUEZ *N* = 445^1^	QUETZALTENANGO *N* = 558^1^	TOTAL *N* = 2,352^1^
**Age (months)**	12.9 (8.8, 17.0)	13.2 (8.6, 18.6)	13.7 (9.1, 18.3)	15.5 (12.2, 19.2)	13.8 (9.6, 18.1)
**Female**	433 (46%)	199 (49%)	225 (51%)	265 (47%)	1,123 (48%)
**Indigenous**	598 (98%)	143 (99%)	161 (88%)	483 (88%)	1,385 (93%)
**Mayan language**	551 (74%)	226 (73%)	0 (0%)	467 (84%)	1,244 (69%)
**Food insecurity**					
**Low**	238 (27%)	75 (20%)	103 (28%)	162 (29%)	579 (26%)
**Moderate**	275 (31%)	140 (36%)	58 (16%)	132 (24%)	605 (28%)
**High**	376 (42%)	169 (44%)	209 (56%)	262 (47%)	1,016 (46%)
**Birthweight (kg)**	2.82 (2.70, 3.18)	2.91 (2.70, 3.18)	3.00 (2.73, 3.20)	3.00 (2.70, 3.20)	2.95 (2.70, 3.18)
**Number of siblings**	2.00 (1.00, 4.00)	2.00 (1.00, 3.00)	2.00 (1.00, 3.00)	2.00 (1.00, 3.00)	2.00 (1.00, 3.00)
**Homeowner**	453 (51%)	203 (52%)	69 (19%)	366 (66%)	1,092 (50%)
**Height‑for‑age z‑score (HAZ)**	–2.87 (–3.43, −2.43)	–2.88 (–3.55, −2.36)	–2.69 (–3.18, −2.31)	–2.94 (–3.62, −2.44)	–2.86 (–3.46, −2.39)
**Height‑for‑age difference (HAD)**	–7.27 (–8.84, −6.10)	–7.45 (–9.39, −5.98)	–7.06 (–8.74, −5.69)	–7.95 (–9.63, −6.51)	–7.43 (–9.19, −6.14)
**Moderate acute malnutrition**	26 (2.8%)	16 (3.9%)	29 (6.6%)	9 (1.6%)	80 (3.4%)
**Severe acute malnutrition**	13 (1.4%)	9 (2.2%)	7 (1.6%)	3 (0.5%)	32 (1.4%)

^1^Median (Q1, Q3); n (%)

The clinical cohort includes 1 child belonging to a department which is not included in the table. Percentages are calculated among non‑missing observations.

WHO IYCF indicators for children upon enrollment, overall and by department, are shown in Table 2. A minority of children met the WHO standards for MDD and MAD upon enrollment, while most children met the standard for MMF. There were some department‑level differences in dietary quality, most notably in Suchitepéquez where a lower percentage of children consumed fruits and vegetables and a greater percentage of children consumed sweet beverages.

**Table 2:** WHO Infant and Young Child Feeding Indicators from a clinical cohort of children with stunting in Guatemala, overall and by department.

WHO INDICATOR	CHIMALTENANGO *N* = 922^1^	SOLOLá *N* = 386^1^	SUCHITEPéQUEZ *N* = 393^1^	QUETZALTENANGO *N* = 556^1^	TOTAL *N* = 2,258^1^
**Minimum dietary diversity**	438 (48%)	134 (35%)	99 (26%)	236 (43%)	907 (41%)
**Minimum meal frequency**	687 (77%)	314 (84%)	222 (63%)	486 (89%)	1,710 (79%)
**Minimum acceptable diet**	389 (43%)	125 (33%)	68 (18%)	232 (42%)	814 (36%)
**Zero fruit or vegetable consumption**	214 (23%)	73 (19%)	173 (44%)	122 (22%)	583 (26%)
**Egg and/or flesh food consumption**	579 (63%)	195 (51%)	174 (44%)	347 (62%)	1,295 (57%)
**Sweet beverage consumption**	476 (52%)	176 (46%)	241 (61%)	207 (37%)	1,101 (49%)
**Unhealthy food consumption**	345 (37%)	120 (31%)	98 (25%)	281 (51%)	844 (37%)

^1^n (%). Only children for whom the first encounter was within the age range of 6–23 months are included. Indicators are based on data from the first visit after 6 months of age.

Of the 2,258 children, 1 child is from a department not included in the table.

**Figure 2:**
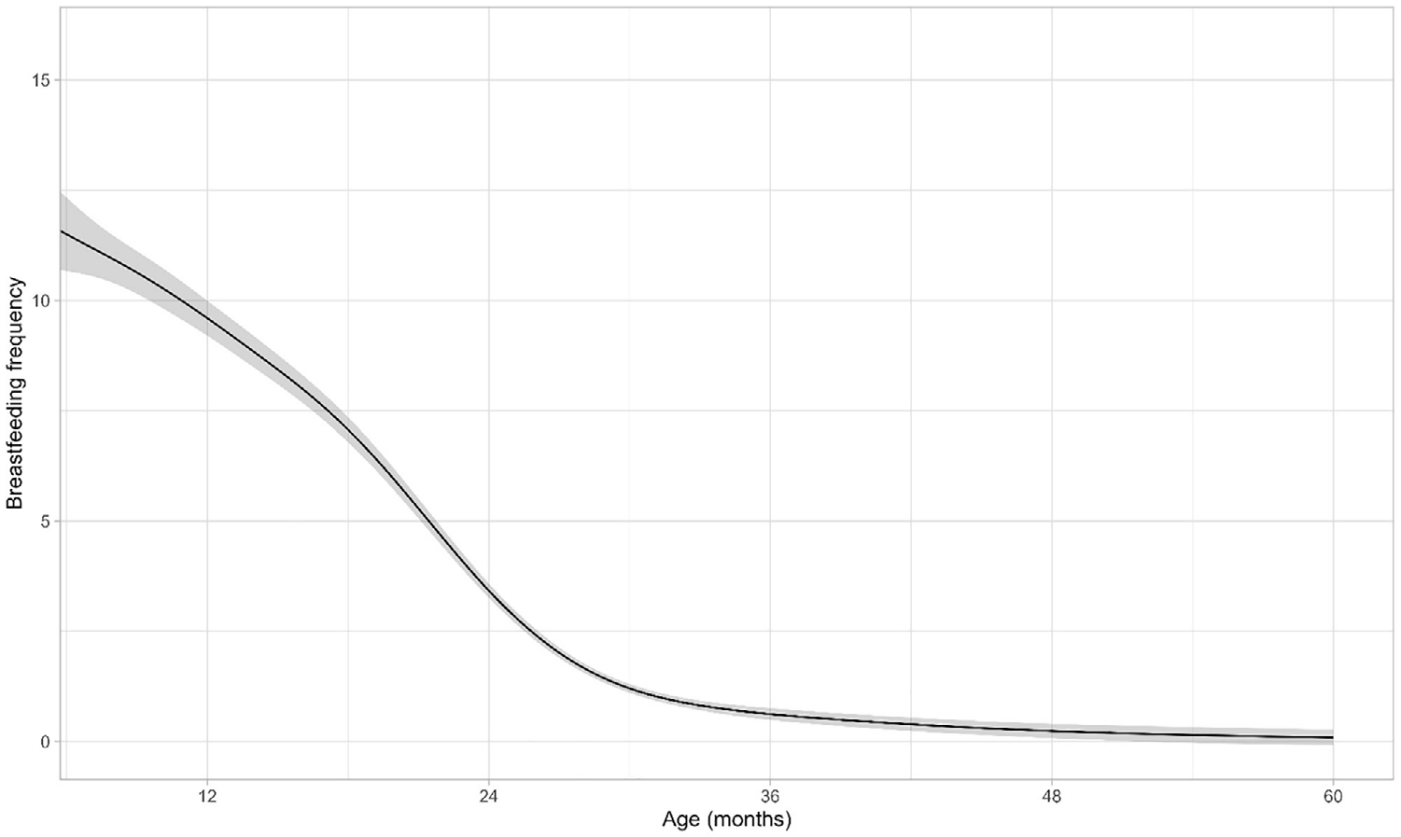
Prediction of breastfeeding frequency from 6–60 months in a clinical cohort of children with stunting in Guatemala using generalized additive mixed modeling (GAMM).

**Figure 3:**
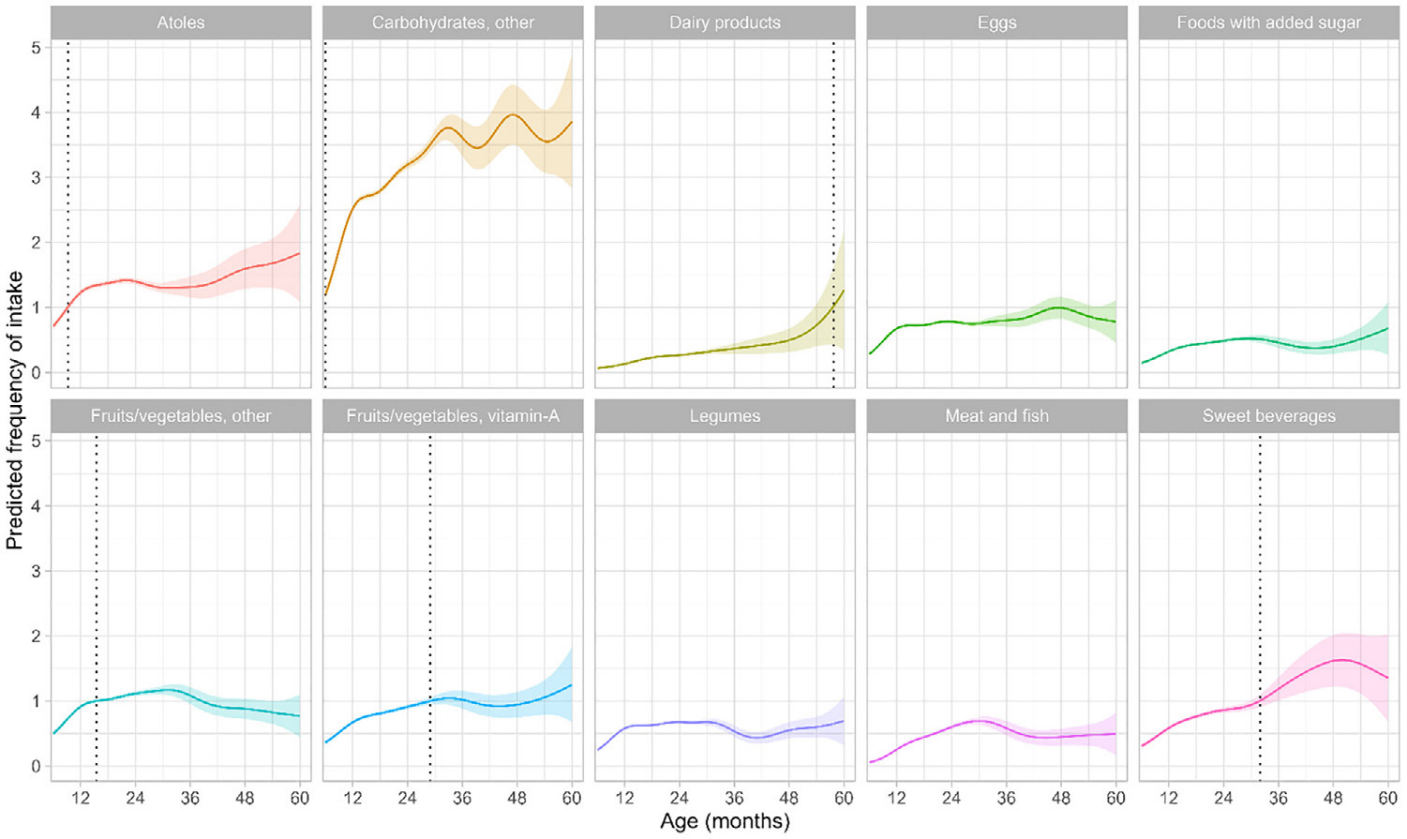
Prediction of intake frequency of different food groups from 6–60 months in a clinical cohort of children with stunting in Guatemala using generalized additive mixed modeling (GAMM).

Modeling of breastfeeding frequency over time is shown in Figure 2. According to model predictions, breastfeeding frequency declined from just over 11 times per day at 6 months to just under four times per day by 24 months and less than once per day by 36 months. Frequency of intake of different food groups is shown in Figure 3, with vertical lines indicating the age at which predicted intake first reached once per day. *Atoles* and other carbohydrates were the only individual food groups whose intake significantly exceeded once per day. However, for the aggregated food groups, frequency of intake of both animal source foods (eggs, meat, fish, or dairy) and fruits and vegetables (of any kind) steadily increased from 6 to 30 months and approached thresholds of 2.5 times per day by that time (Supplementary Figure 1). GAMM modeling of dietary diversity over age by department and overall is shown in Supplementary Figure 2. In general, dietary diversity increased from 6–12 months of age and tapered off slightly at 30–36 months of age.

**Figure 4:**
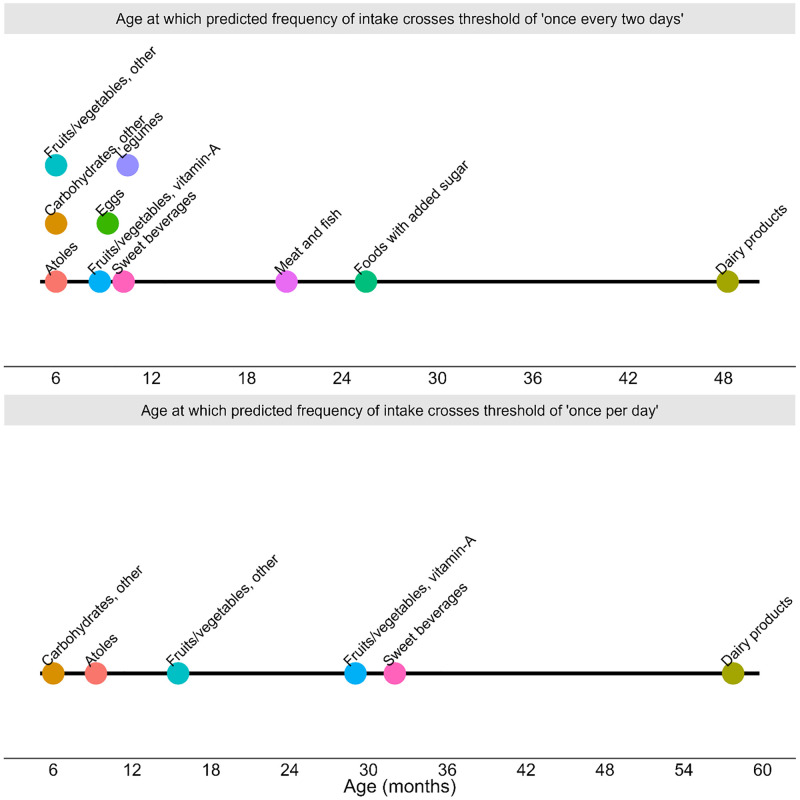
Age at which predicted frequency of intake of different food groups crossed thresholds of once every two days and once per day in a clinical cohort of children with stunting in Guatemala.

Figure 4 shows the relative order in which frequency of intake for individual food groups reached thresholds of “once every two days” and “once per day.” By 12 months of age, only *atoles* and other carbohydrates reached the threshold of daily consumption, and by 36 months, the only additional foods to reach that threshold were vitamin A‑rich fruits and vegetables, other fruits and vegetables, and sweet beverages. A greater variety of foods, including legumes eggs, vitamin A‑rich fruits and vegetables, other fruits and vegetables, and sweet beverages, reached the threshold of “once every two days” by 12 months. However, animal flesh foods lagged, not reaching that threshold until approximately 20 months.

**Figure 5:**
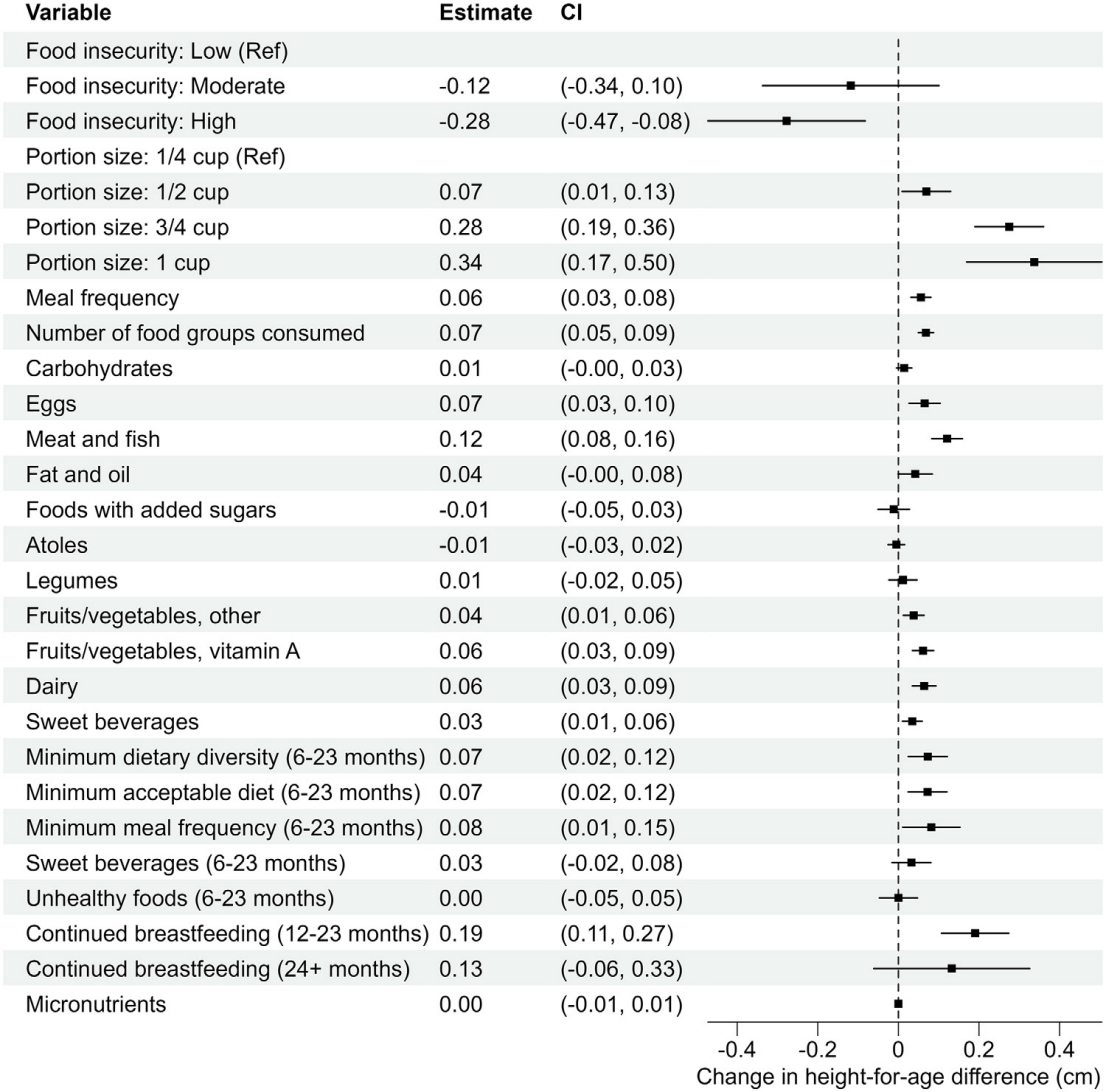
Associations between dietary and breastfeeding factors and linear growth (cm) in a clinical cohort of children with stunting in Guatemala using linear mixed effects modeling and controlling for age, sex, and birthweight.

The results of LME modeling demonstrated that the factors most strongly associated with linear growth were food insecurity, portion size, and continued breastfeeding. As shown in Figure 5, higher degrees of food insecurity tended to be associated with poorer linear growth and larger portion sizes with better growth. Continued breastfeeding between 12 and 23 months of age was associated with better linear growth, but the relationship lessened and became non‑significant after 24 months of age. Among individual food groups, the consumption of eggs and animal flesh foods had the strongest relationships with growth, followed by vitamin A‑rich fruits and vegetables, and other fruits and vegetables. The WHO IYCF indicators MDD, MMF, and MAD, when met, were also associated with better growth. Marginal plots demonstrated that the relationship between continued breastfeeding and linear growth switched from positive to negative at around 30 months of age and that as children got older, larger portion sizes and meeting minimum meal frequency became more strongly associated with linear growth (Supplementary Figure 3).

## Discussion

We analyzed clinical data from a clinical cohort of 2,352 children with stunting from rural Guatemala who were enrolled in a nutrition program that provides growth monitoring, nutrition counseling, short‑term food and micronutrient support, and medical support. The objective of our study was to characterize dietary patterns and to identify factors positively or negatively associated with linear growth in this cohort. We opted to conduct a chart review given that longitudinal dietary and growth data from a large clinical cohort of stunted children would not be available from demographic health surveys and other public sources. Because of the interventions provided by the nutrition program, growth trajectories may have improved over time more than they would have in a purely observational study.

We found in our analysis that most children did not meet WHO standards for dietary adequacy upon enrollment. Dietary intakes were predominantly carbohydrate‑based, though they also contained some fruits, vegetables, and animal source foods. Eggs, legumes, and animal flesh foods individually never reached a predicted frequency of intake of “once per day.” In LME modeling, the factors most strongly associated with linear growth were food insecurity (–), portion size (+), and continued breastfeeding (+). The WHO indicators MDD MMF, and MAD were positively associated with linear growth, as were a variety of food groups, of which the strongest associations were found for animal flesh foods and eggs.

This study has limitations. As previously mentioned, it is not purely observational in the sense that children enrolled in the nutrition program received interventions throughout the course of the program. Thus, dietary quality and growth may have improved over time more than they would have in a purely observational study. Social desirability bias may have influenced intake frequency reporting, leading to inflated reported intake frequencies of foods perceived to be healthy. Additionally, diet recall techniques may have differed among nutrition technicians administering the program in different regions. Strengths of the study include a large sample size and the use of repeated measures longitudinal data with multiple dietary and anthropometric measurements per child. Through predictive modeling, we were able to show how breastfeeding frequency and dietary intake changed across age for children with stunting.

We briefly compare our findings here to some of the existing literature. In a recent study of dietary patterns of preschool‑age Maya children from Guatemala, 54% of whom were stunted, two distinct dietary patterns were identified using k‑means clustering, one grain‑based (*n* = 79) and the other rich in dairy, poultry, and corn (*n* = 76) [11]. In the grain‑based diet, 73% of calories came from carbohydrate‑based foods (excluding fruits) versus 47% in the more varied diet. However, stunting prevalence was not significantly different between the two groups. By contrast, we found carbohydrates to be the predominant food source for Guatemalan children with stunting in this clinical cohort, with *atoles*, sweet beverages, and other carbohydrates reaching the highest frequency of intake of all the food groups while key sources of protein including animal source foods, legumes and eggs never reached an intake frequency of “once per day” throughout the first 60 months of age.

Additionally, a cross‑sectional study of preschool‑aged children in Mexico used principal component analysis (PCA) to characterize dietary patterns [12]. Two dietary patterns emerged that were associated with higher stunting prevalence: one characterized by high intake of fruits and vegetables, with few other foods consumed, and the other characterized by corn‑based and other traditional foods. A pattern characterized by higher intakes of meat, fat, and sugar was associated with lower stunting prevalence but higher overweight prevalence. Consistent with this study, we found that carbohydrates and fruits and vegetables were the only food groups to be consumed daily by 2 years of age among stunted children. However, while we did find that animal flesh foods and sweet beverages were positively associated with linear growth, we also found that fruits and vegetables had positive associations. The fact that most foods were positively associated with linear growth in our analysis, and that portion size was found to be the most important predictor, may indicate that total energy intake played an important role in linear growth.

Regarding WHO IYCF recommendations, children in this clinical cohort generally met the recommendation for continued breastfeeding through 2 years of age. Given our decision to exclude records prior to 6 months of age, limited data were available to estimate the age of introduction of different complementary foods. However, the available data do suggest that by 6 months of age, most children were consuming *atoles*, other carbohydrates, and some fruits and vegetables. Most children were not meeting MDD or MAD upon enrollment, and a significant proportion were eating unhealthy foods and beverages. However, most of them did meet MMF. According to predictive modeling, children generally met the WHO recommendation to consume fruits and vegetables and animal source foods daily, but not until 7.5 and 11 months, respectively, and legumes were not frequently consumed as recommended.

The findings of this study suggest that the nutritional focus of interventions for children from this clinical cohort should be on adequate dietary diversity, earlier introduction and higher frequency of intake of certain food groups, particularly eggs, legumes, animal flesh foods, and dairy products, age‑appropriate portion sizes, and continued breastfeeding through 2 years of age. Given that most children in the cohort already meet this latter recommendation, nutrition messaging may also consider our finding that breastfeeding after two and a half years of age seems to inhibit linear growth. Additionally, nutrition programs may consider providing some of the less frequently consumed food groups, particularly those that tend to be cost‑prohibitive for families. On a policy level, measures to address food insecurity are needed as our study confirmed that food insecurity was an important negative predictor of linear growth. It is unclear to what degree these findings generalize to broader populations of children with stunting in Guatemala, Latin America, and the rest of the world. Important areas for future research include cultural practices pertaining to complementary feeding, effects of distinct complementary feeding patterns on gut microbial health, and the relationship between quality of dietary intake and cognitive development.

## Data Availability

Data described in the manuscript, code book, and analytic code will be made publicly available at: Alajajian, Stephen, 2026, “Breastfeeding and complementary feeding practices of children with stunting in Guatemala,” https://doi.org/10.7910/DVN/X9E1T7, Harvard Dataverse.

## Additional File

The additional file for this article can be found as follows:

Supplementary Material. Supplementary Table 1, Supplementary Figures 1 to 3 and Supplementary Maps.
