# Supplementary material for: Breastfeeding and Complementary Feeding Practices of Children with Stunting in Guatemala
Source: Ann Glob Health. 2026 Aug 6;92(1):78. doi: 10.5334/aogh.5266 (PMC13450269; doi:10.5334/aogh.5266)
Supplement: Supplementary file 1 [file agh-92-1-5266-s1.pdf]

Supplementary Materials

Supplementary Table 1. Missing data counts

	variable	n_miss	pct_miss
1	exclusive_bf	19476	100
2	continued_bf	7061	36.25
3	ethnicity	7045	36.17
4	language	4293	22.04
5	recuento_veces_mamar	4108	21.09
6	mmf	4022	20.65
7	recuento_veces_mamar_noche_leg	3997	20.52
8	mad	3841	19.72
9	mdd	3817	19.6
10	sweet_bev	3707	19.03
11	zero_fv	3703	19.01
12	egg_meat	3703	19.01
13	unhealthy_foods	3702	19.01
14	anthro_wflz	2123	10.9
15	anthro_haz	2107	10.82
16	anthro_had	2107	10.82
17	anthro_waz	2034	10.44
18	intake_number_siblings	2019	10.37
19	anthro_height_cm	2003	10.28
20	anthro_weight_kg	1988	10.21
21	intake_owns_house	1230	6.32
22	anthro_birth_weight_kg	1126	5.78
23	food_insecurity	1090	5.6
24	intake_material_techo_leg	1000	5.13
25	anthro_gest_age_delivery	849	4.36
26	recuento_portion_size	294	1.51
27	recuento_veces_comer_leg	257	1.32
28	fg_consumed	171	0.88
29	recuento_currently_bf	147	0.75
30	recuento_solido_semi_suave_leg	120	0.62
31	recuento_milk_formula	20	0.1
32	first_valid_wflz	19	0.1
33	desnut_aguda_mod	19	0.1
34	desnut_aguda_sev	19	0.1
35	recuento_sweet_bev_freq	9	0.05
36	recuento_drink_mix	8	0.04
37	recuento_gruel	8	0.04
38	recuento_animal_source	8	0.04
39	recuento_carbos	7	0.04
40	recuento_foods_refined_added_sugars	7	0.04
41	recuento_water_potable	7	0.04
42	recuento_coffee_tea	5	0.03
43	recuento_eggs	4	0.02
44	recuento_condiments	3	0.02

45	recuento_foods_enriched_fats	3	0.02
46	recuento_legumes	3	0.02
47	recuento_dairy	3	0.02
48	recuento_any_fv	3	0.02
49	recuento_broth	2	0.01
50	recuento_flesh	2	0.01
51	recuento_fv_other	2	0.01
52	recuento_milk_animal	2	0.01
53	recuento_milk_cheese	1	0.01
54	recuento_fv_vitamin_a	1	0.01
55	recuento_juice	1	0.01
56	study_id	0	0
57	encounter_datetime	0	0
58	edad_meses	0	0
59	encounter_location	0	0
60	gender	0	0
61	recuento_soda_pop	0	0
62	recuento_soup_mix	0	0
63	recuento_water_with_sugar	0	0
64	department	0	0
65	first_valid_haz	0	0
66	gravedad_restraso	0	0

Supplementary Figure 1. Consumption frequency of differnet food groups

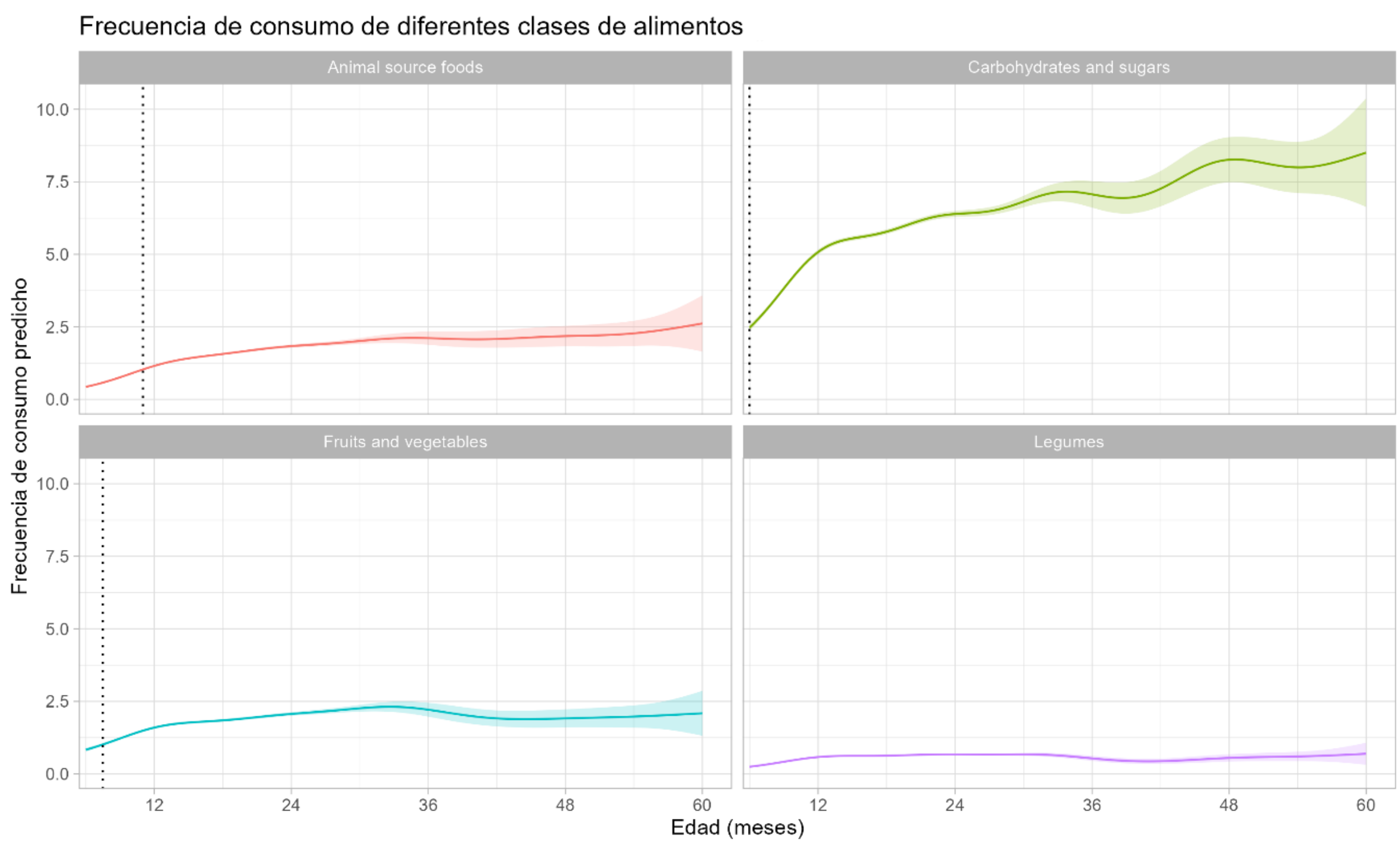

Supplementary Figure 2. Diet diversity over age

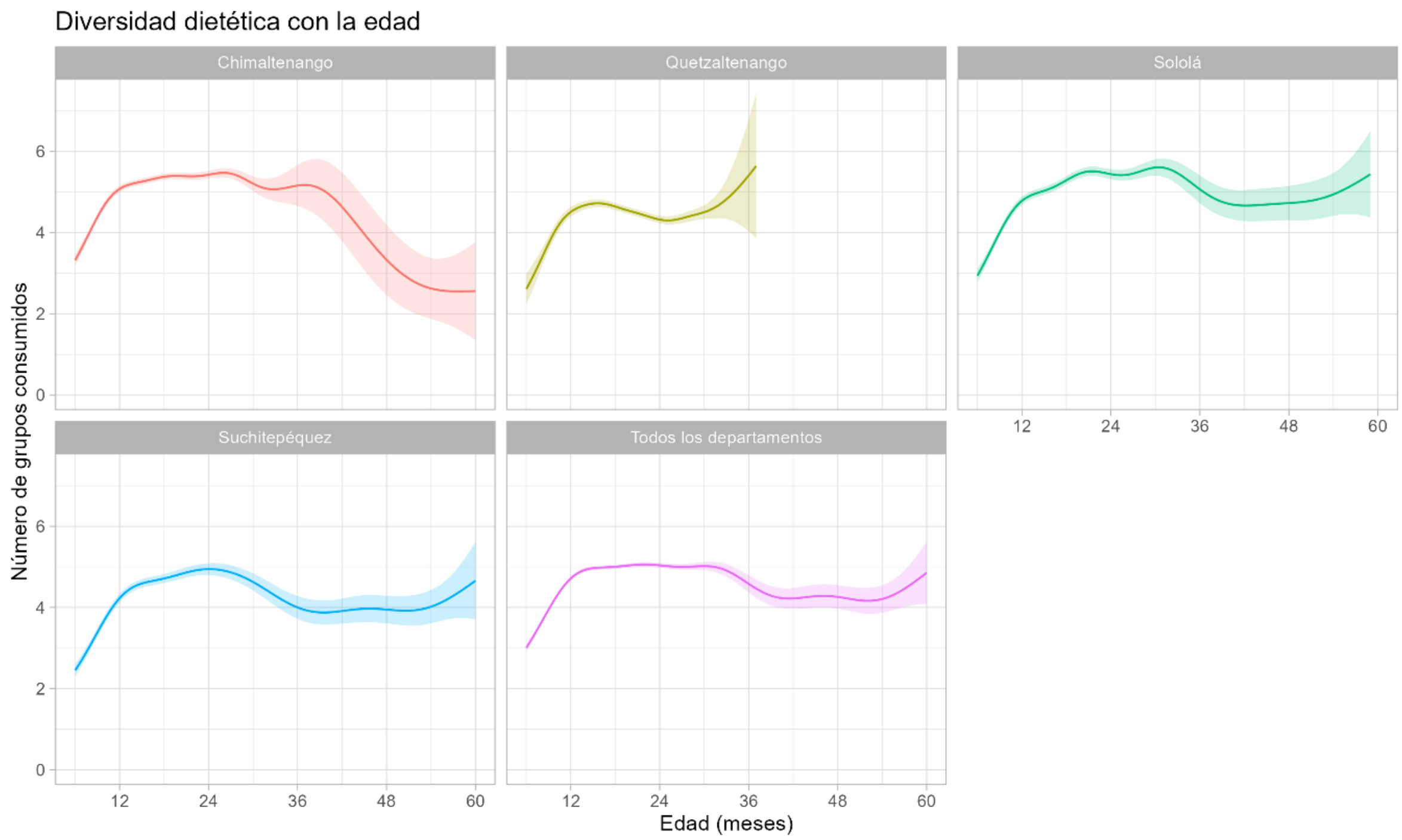

Supplementary Figure 3. Marginal effects over time

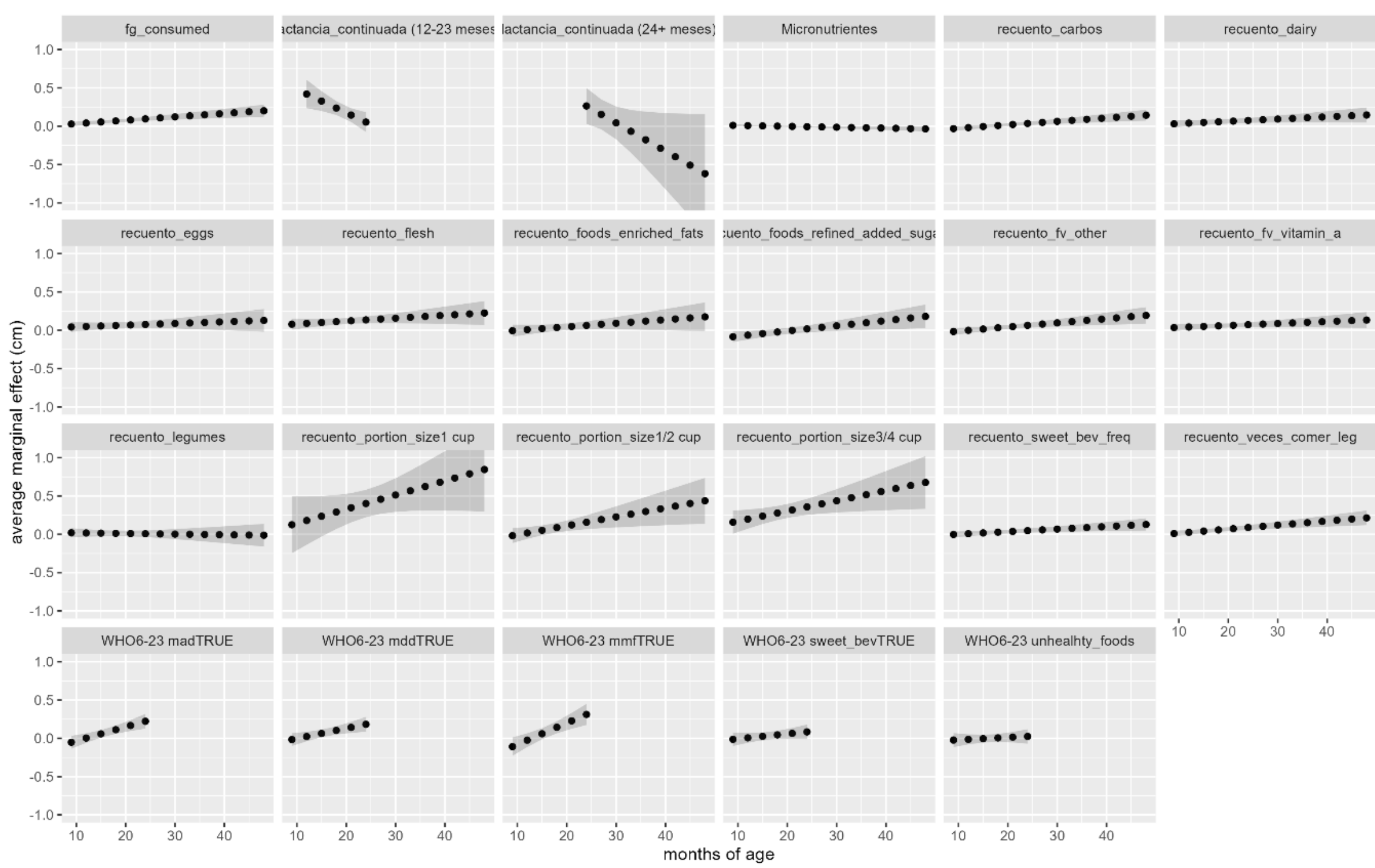

Supplementary Maps

- Prevalences take into account all observations from all individuals
- Names of villages removed

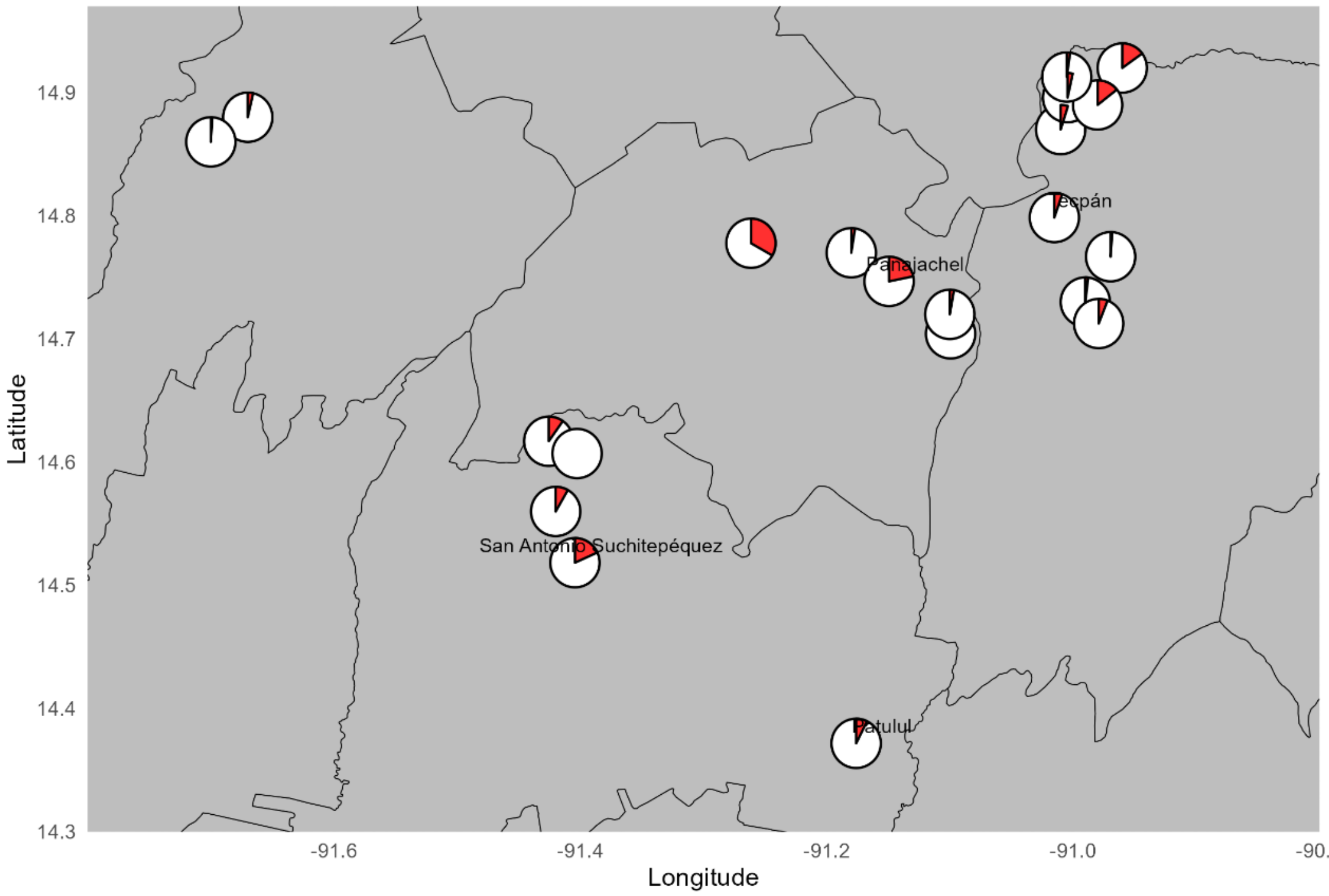

A Acute malnutrition prevalence

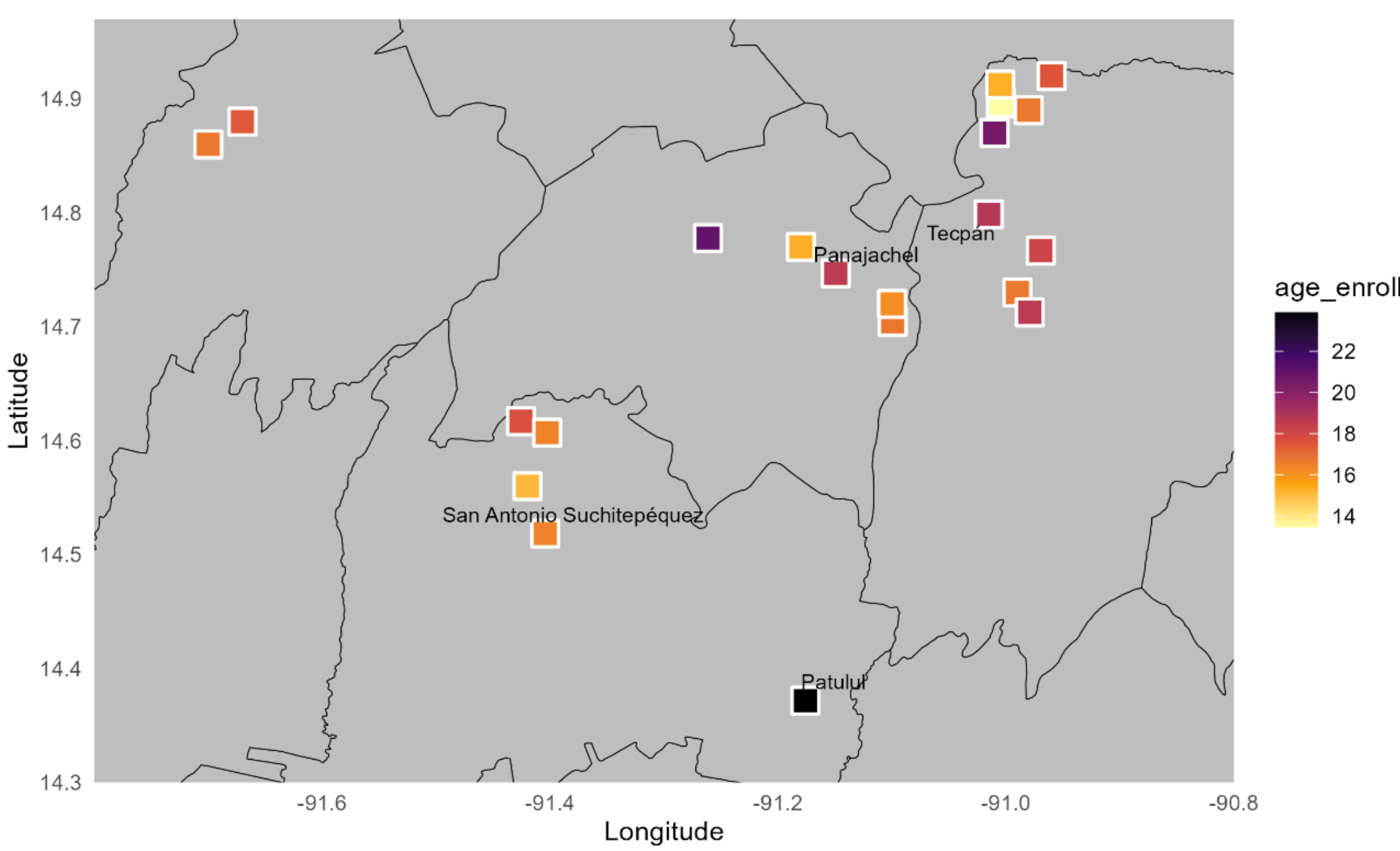

B Median age upon enrollment by location

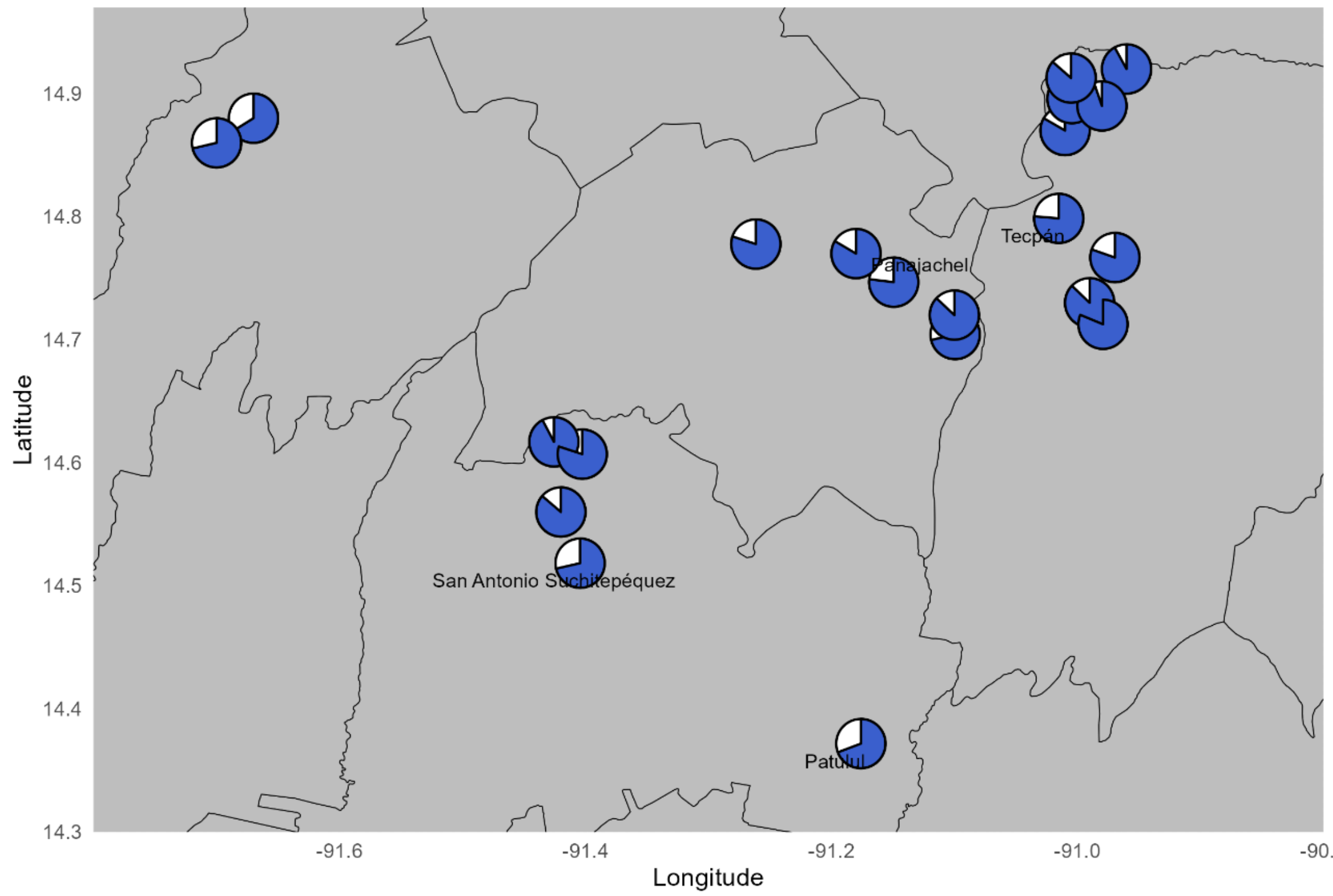

C Prevalence of continued breastfeeding

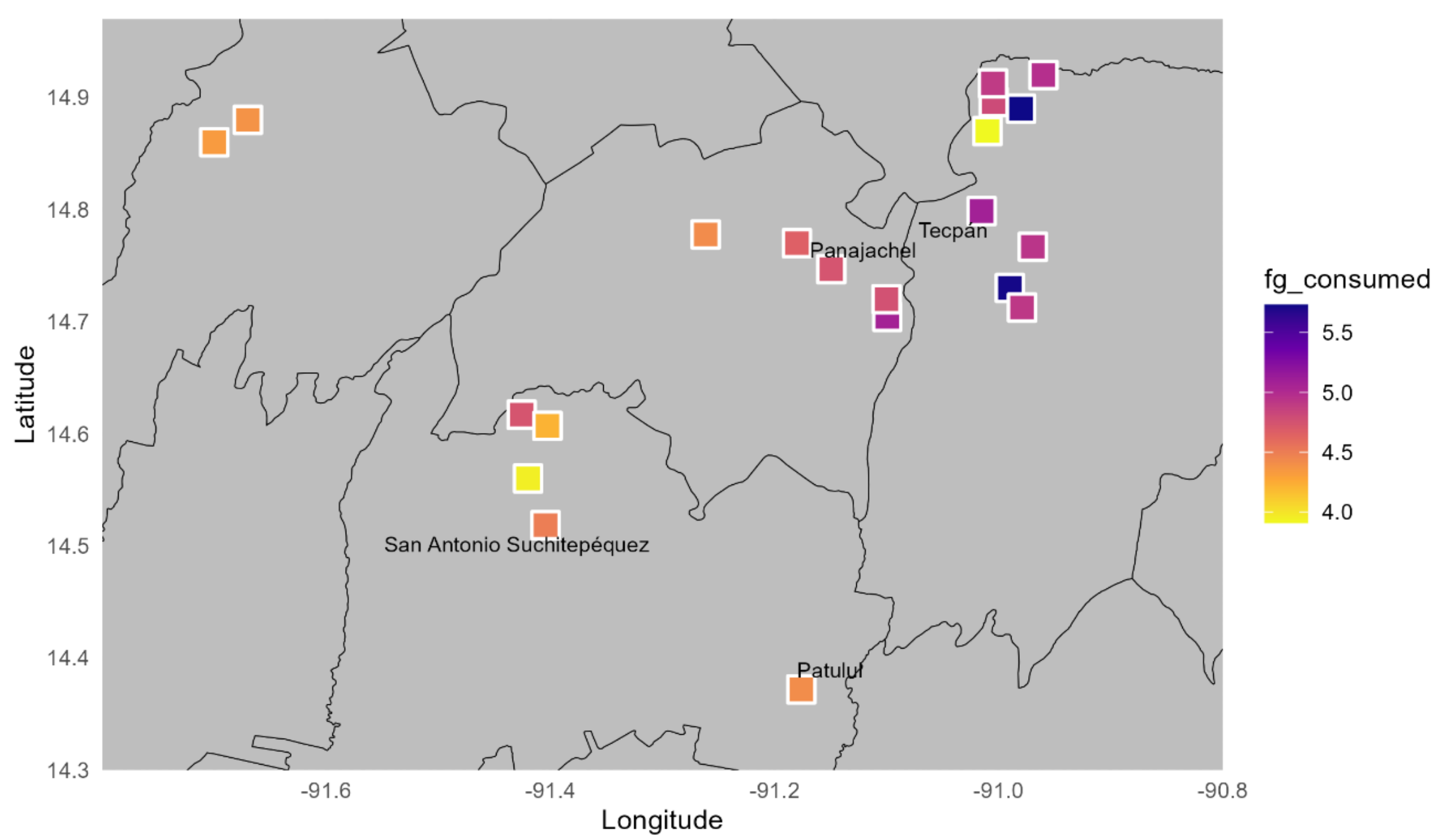

D Average number of food groups consumed

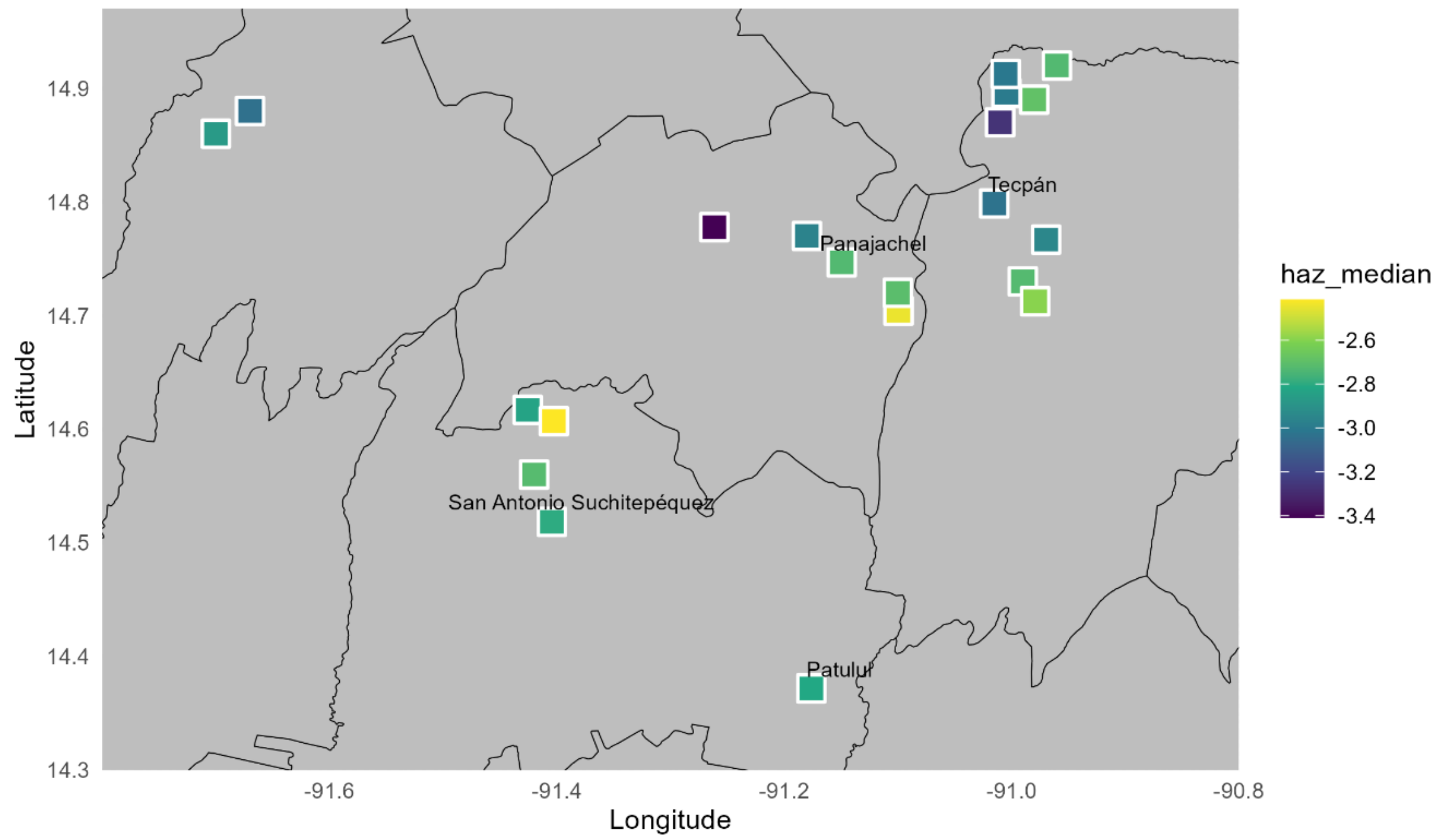

E Median height-for-age z-score

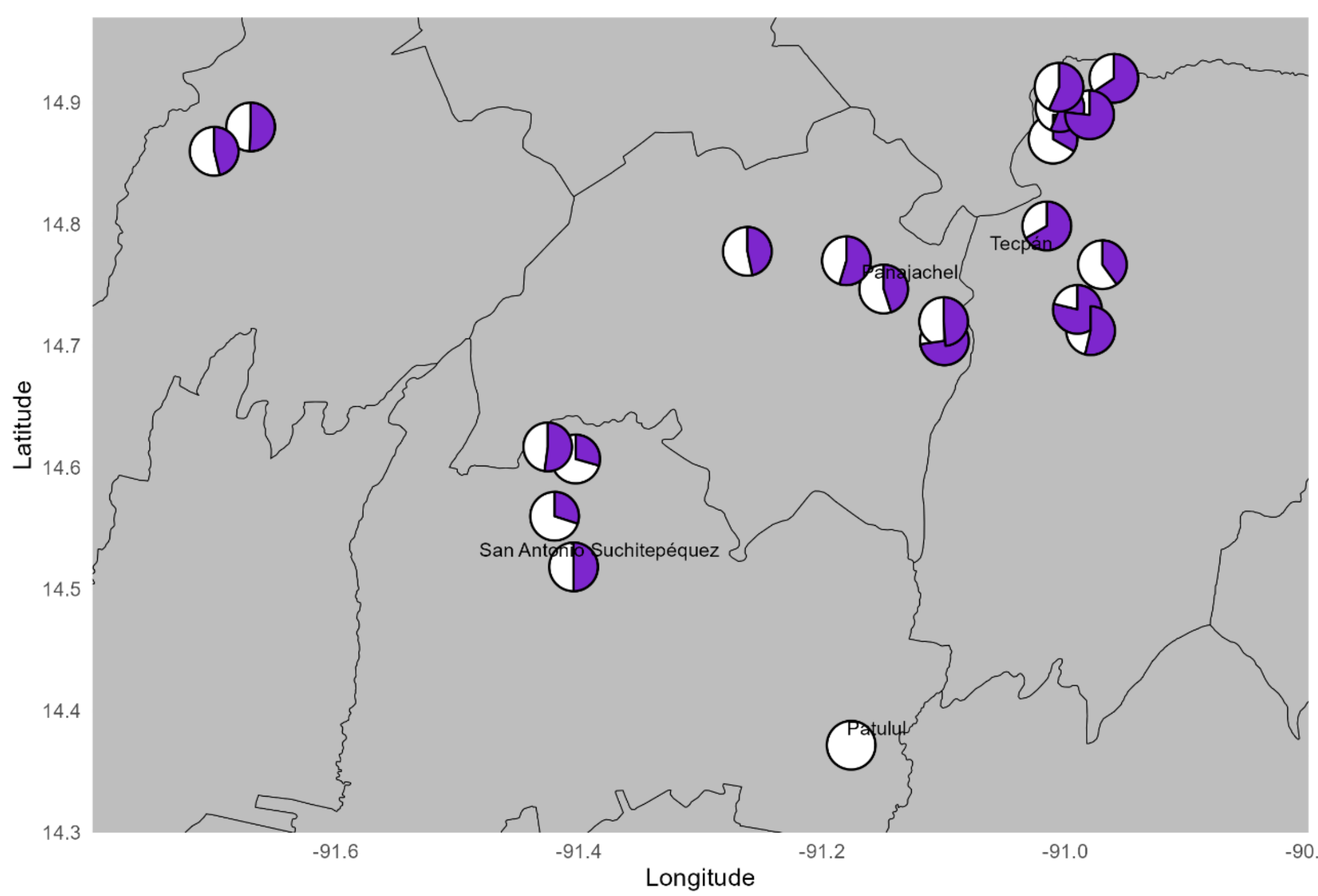

F Prevalence of meeting minimum acceptable diet

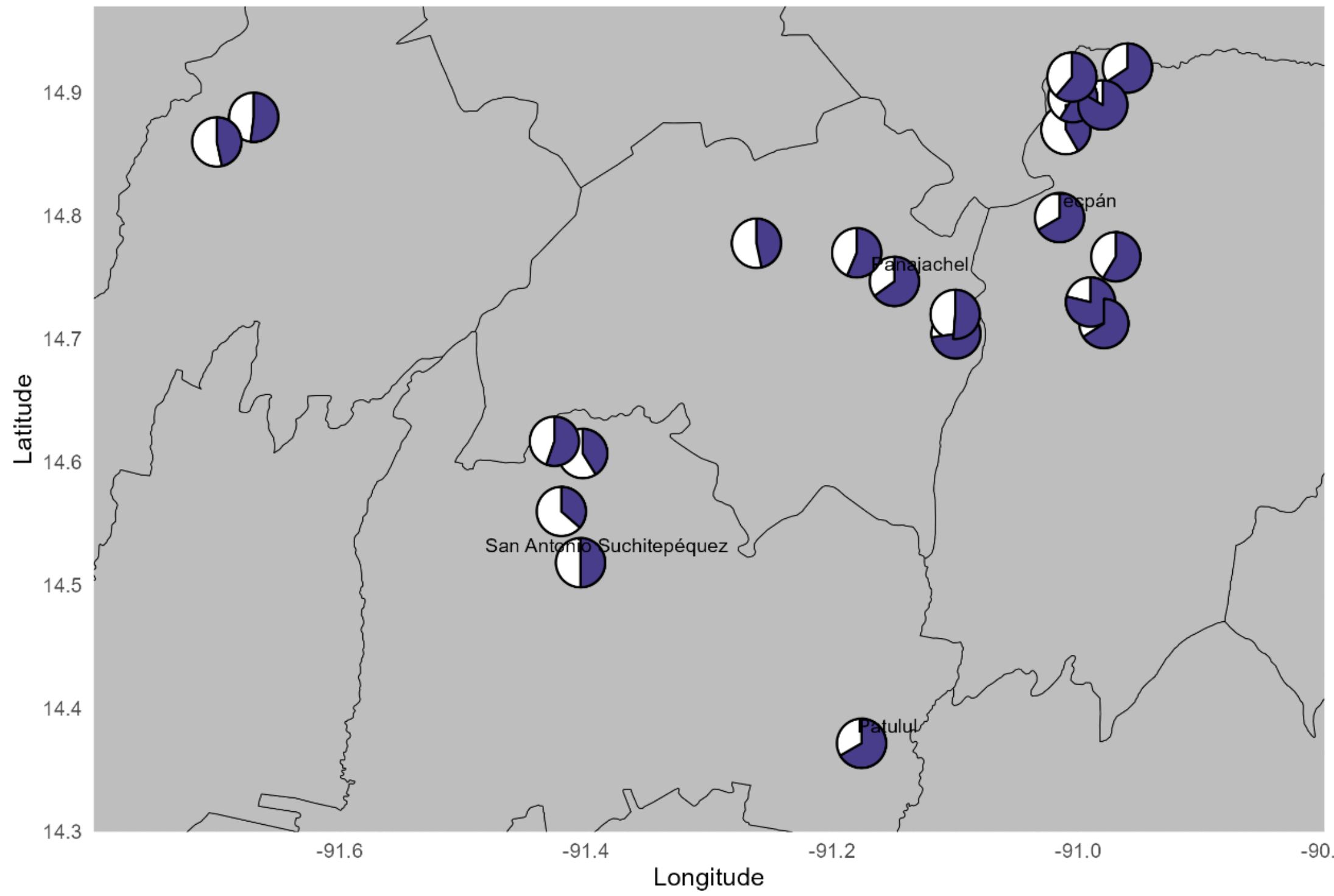

G Prevalence of meeting minimum diet diversity

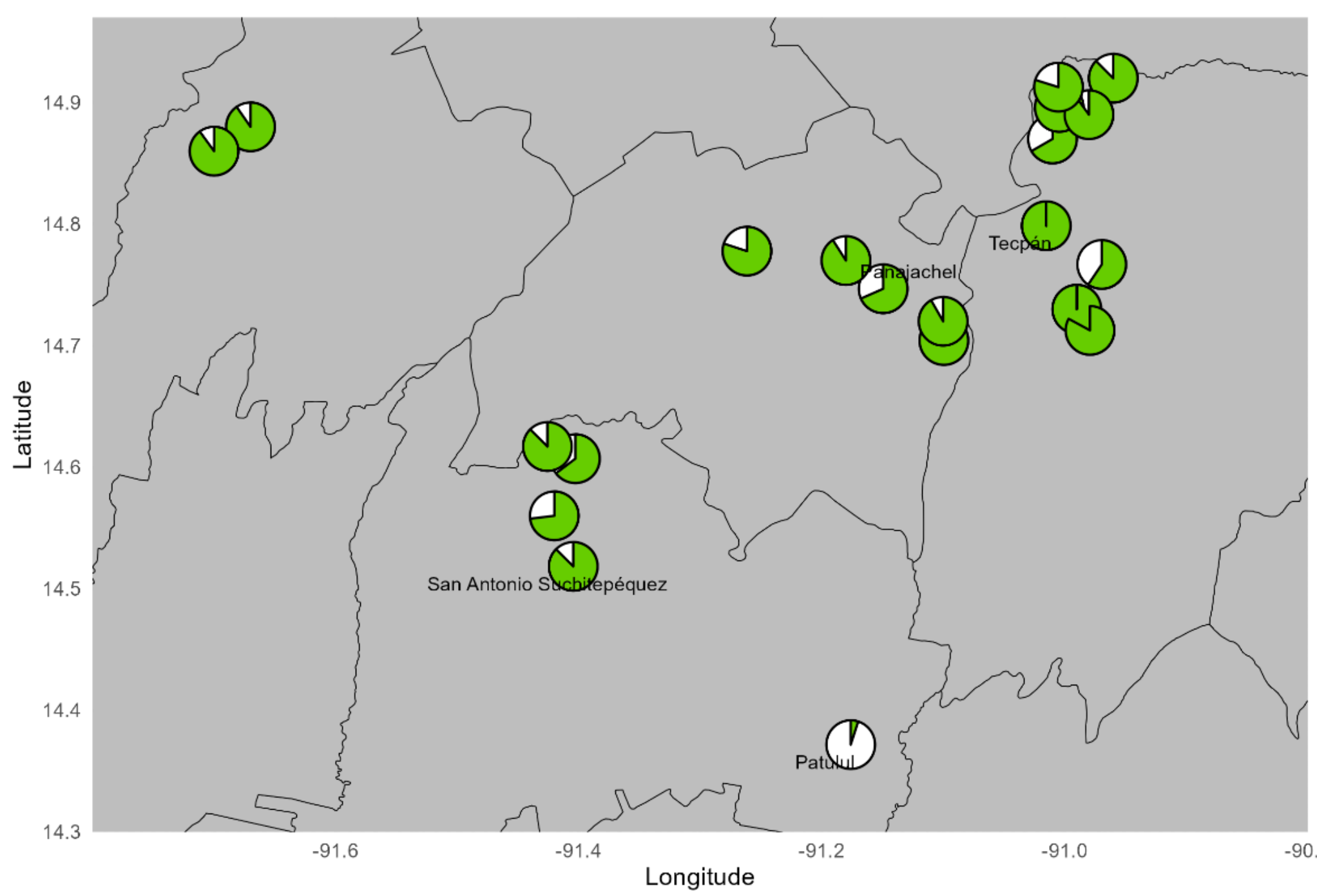

H Prevalence of meeting minimum meal frequency

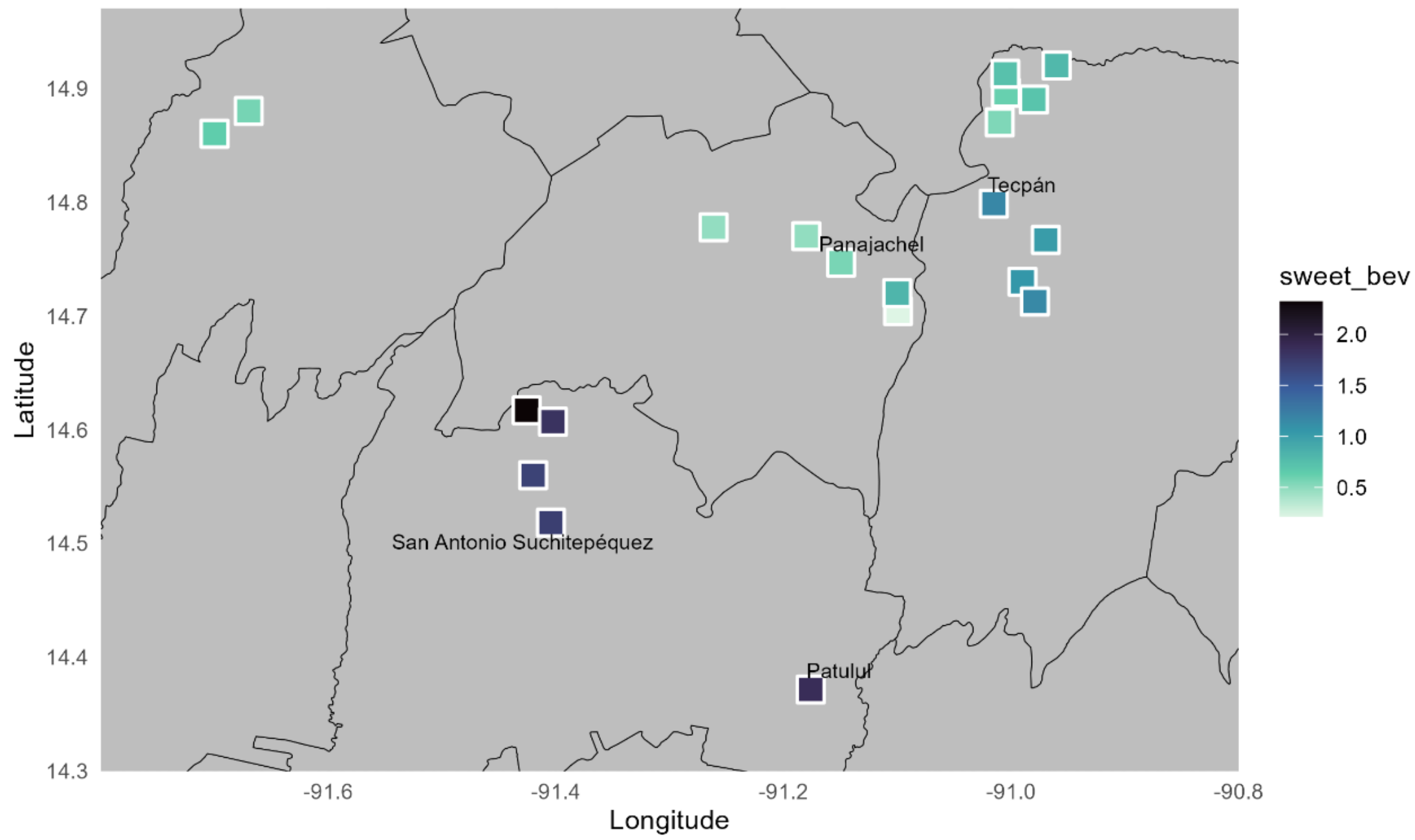

I Average number of sweet beverages consumed

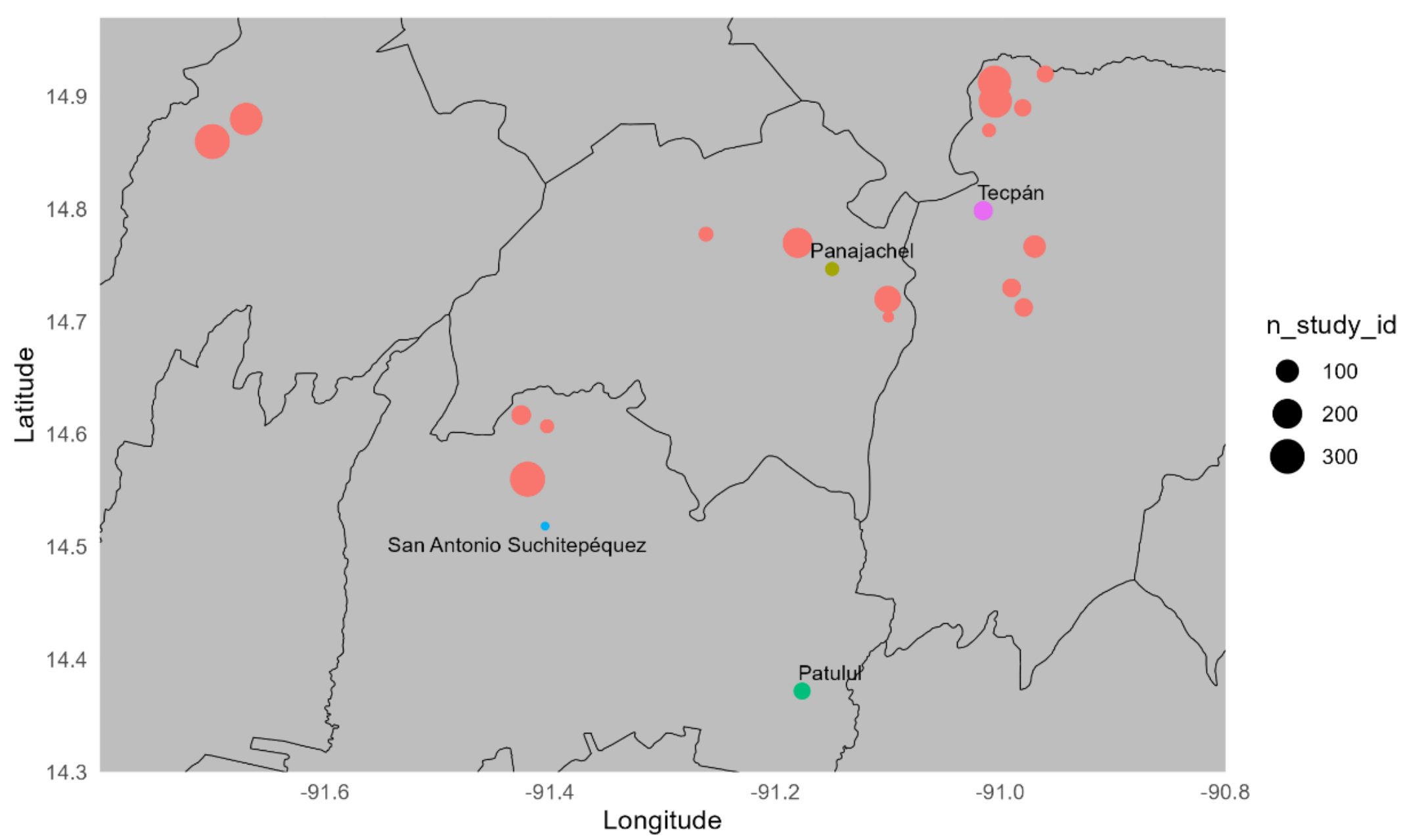

J Number of children
